# Uncovering Cryptic Parasitoid Diversity in *Horismenus* (Chalcidoidea, Eulophidae)

**DOI:** 10.1371/journal.pone.0136063

**Published:** 2015-09-09

**Authors:** Sarah G. Kenyon, Sven Buerki, Christer Hansson, Nadir Alvarez, Betty Benrey

**Affiliations:** 1 Laboratory of Evolutionary Entomology, Institute of Biology, University of Neuchatel, Neuchâtel, Switzerland; 2 Department of Life Sciences, Natural History Museum, Cromwell Road, London, United Kingdom; 3 Museum of Biology (Entomology), Lund University, Lund, Sweden; 4 Department of Ecology and Evolution, Biophore Dorigny, University of Lausanne, Lausanne, Switzerland; Portland State University, UNITED STATES

## Abstract

*Horismenus* parasitoids are an abundant and understudied group of eulophid wasps found mainly in the New World. Recent surveys based on morphological analyses in Costa Rica have quadrupled the number of named taxa, with more than 400 species described so far. This recent revision suggests that there is still a vast number of unknown species to be identified. As *Horismenus* wasps have been widely described as parasitoids of insect pests associated with crop plants, it is of high importance to properly establish the extant diversity of the genus, in order to provide biological control practitioners with an exhaustive catalog of putative control agents. In this study, we first collected *Horismenus* wasps from wild *Phaseolus* bean seeds in Central Mexico and Arizona to assess the genetic relatedness of three morphologically distinct species with overlapping host and geographical ranges. Sequence data from two nuclear and two mitochondrial gene regions uncovered three cryptic species within each of the three focal species (i.e., *H*. *missouriensis*, *H*. *depressus* and *H*. *butcheri*). The monophyly of each cryptic group is statistically supported (except in two of them represented by one single tip in which monophyly cannot be tested). The phylogenetic reconstruction is discussed with respect to differences between gene regions as well as likely reasons for the differences in variability between species.

## Introduction

As a protein-rich pulse, *Phaseolus* beans are an important crop plant worldwide. The common bean, *P*. *vulgaris*, originated [1, 2] and was domesticated [3–5] in Mexico and now is cultivated worldwide. Mexico is also host to a number of other *Phaseolus* beans [6]. Some, such as the scarlet runner bean (*P*. *coccineus*), have their origin in the region and others, such as the lima bean (*P*. *lunatus*), have one of their early diversification centers here [6]. The mature wild beans of these species are attacked by bruchine beetles, largely in the genus *Acanthoscelides* and *Zabrotes* [7–10]. These bruchine beetles are in turn, attacked by a suite of parasitoid wasps. The most abundant wasps are within the genus *Horismenus*. All this makes for a rich environment to explore the native associations between these beans, their pests and the natural enemies of these pests in both wild and agricultural settings.

**Fig 1:**
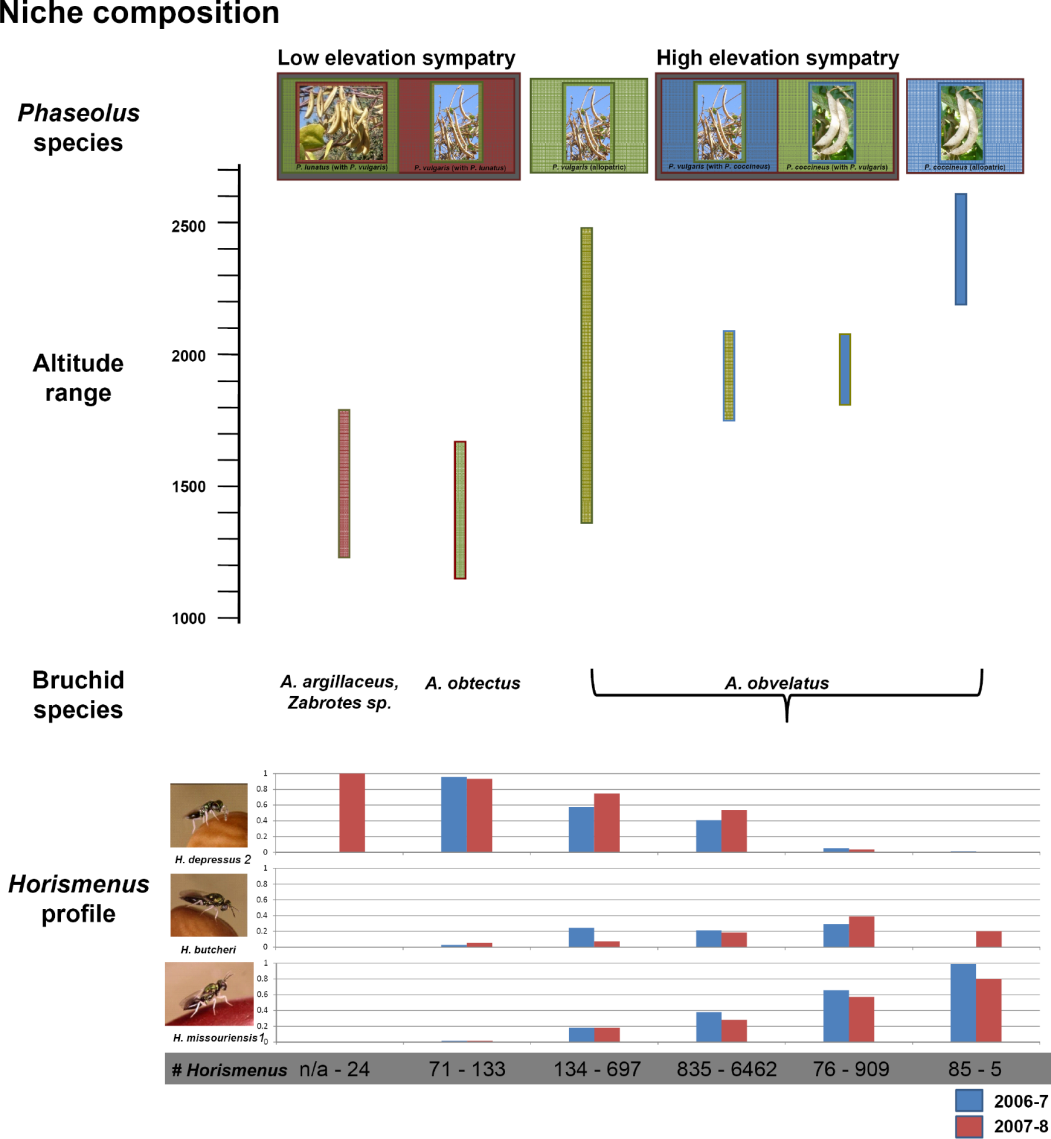
Tritrophic relationships. *Phaseolus species*: Depicted at the top of the figure are three wild bean species sorted into categories based on the other beans found in their immediate environment. *Phaseolus lunatus* with a red background is found on the far left with its sympatric partner, *P*. *vulgaris* in green. To their right, *P*. *vulgaris* (again in green) is depicted alone as it is found at median altitudes to be in allopatry. This is followed by *P*. *vulgaris* (in green) and *P*. *coccineus* (in blue) found in sympatry and finally *P*. *coccineus* (in blue) alone to represent where it is found in allopatry. *Altitude range*: Directly beneath these is an altitude measure on the y-axis. The bars indicate the range of altitudes within which each of the above categories was collected. *Bruchine species*: Beneath this are the most common bruchine beetle speces that attack each one of the *Phaseolus* categories. *Horismenus profile*: Photos on the left identify the three species of described *Horismenus* wasp found emerging from bean seeds. Blue bars indicate emergence from the first field season (2006–7) and pink bars indicate emergence from the second field season (2007–8). Numbers below in gray are the number of emerging wasps from that category for that season. The pink and blue bars are the proportions of each wasp species to emerge from that category in each season and will all add to 1.

In a previous study, three species of *Horismenus* wasps that attack beetles within seeds of wild *Phaseolus* species were identified in Central Mexico [11]. The bean species are distributed along an altitudinal gradient with *P*. *lunatus* at the lowest altitudes in humid environments between sea level and 1600m. *Phaseolus vulgaris* is found along the whole range in this area of Mexico, though it is most common in Oak woodland or disturbed areas between 1000 and 2000m. *Phaseolus coccineus* is found in Pine Oak woodland upwards of 1700m [12]. The study area is on the southern edge of the Mexican altiplano (high plain). In this region, the altitude drops quickly from over 2200m to less than 1000m, easily accommodating the ranges of the three bean species. This altitudinal gradient found in the beans appears to be mirrored by both the beetles and the wasps [13]. Ecological surveys ([13], reviewed in Fig 1) indicate that distributions of three *Horismenus* species associated with *Phaseolus*’ natural enemies, viz., *H*. *missouriensis*, *H*. *depressus* and *H*. *butcheri* show distinct but overlapping niches (see Materials and Methods). The spatial overlap between bean species and bruchine hosts, together with the great morphological similarity among the *Horismenus* species, has made the study of the ecology and population genetics of this tritrophic system difficult. Thus, it becomes crucial to resolve unambiguously their taxonomic status. To do this, we used a general species delimitation method (here the Bayesian Poisson tree process or bPTP; [14]) applied to phylogenetic placement. This method has been shown to outcompete most other approaches to define species in a phylogenetic context (e.g. simple sequence similarity thresholds or OUT-picking and the General Mixed Yule coalescence model) and has the advantage of not requiring an ultrametric tree. The latter aspect is very important in the case of this study since we lack of fossils of this group of parasitoid wasps to calibrate the phylogenetic framework.

In this study, we used a combination of sampling techniques (of the three species of *Horismenus* occurring in the study area together with additional species from North and Central America) and molecular methods to establish the identities and relationships among *Horismenus* wasps based on a bPTP approach applied to a phylogenetic framework inferred from four DNA regions. Collecting adequate records of bean/beetle/wasp combinations is also necessary to describe associations between trophic levels and provide an accurate background for making educated inferences regarding genetic and theoretical work.

## Materials and Methods

### Sampling of *Horismenus* wasps

Mature bean pods of *Phaseolus coccineus*, *P*. *lunatus* and *P*. *vulgaris* were collected in the winter dry seasons in central Mexico between December 2004 and March 2008. Individual *Horismenus* wasps were identified and sexed using Hansson *et al*.’s [11] key. These included three species: *H*. *butcheri* (HB), *H*. *depressus* (HD) and *H*. *missouriensis* (HM). A representative sample of male and female wasps from different geographic locations and bean species was selected to represent the range of habitat types where each species was found (69 HB, 14 HD and 26 HM). These emerged from 24 different locations across central Mexico (Table 1): 10 locations with *P*. *coccineus*, 19 with *P*. *vulgaris*, and 1 with *P*. *lunatus*. Six of these locations had sympatric populations of *P*. *vulgaris* and *P*. *coccineus* (CVC, MALS, SJS, TEMS, TEP and TYN), with wasps emerging from both species. Once collected, beans were removed from the pods, separated by species, location and date of collection, placed in ventilated plastic containers and stored at 26–7°C at 70% RH in a 14/10 L/D cycle in a Conviron G30 incubator. Containers were checked every 1–4 days and emerging insects were collected and transferred to 96% EtOH after death.

**Table 1:** Locations sampled in Central Mexico. Locations sampled in Central Mexico with their geographical coordinates. Locations where sampled for bean species (*Phaseolus vulgaris*, *P*.*coccineus* and P. *lunatus*) and *Horismenus* parasitoid emergence. Table shows site code for each location, state, bean species present at each location (symp(vc) = sympatric populations of *P*. *vulgaris* and *P*. *coccineus*; symp(vl) = sympatric populations of *P*. *vulgaris* and *P*. *lunatus*; allop(c) = allopatric populations of *P*. *coccineus*; allop(v) = allopatric populations of *P*. *vulgaris* and allop (cult,v) = allopatric population of cultivated *P*. *vulgaris*), bean species from which parasitoids emerged, geographical coordinates (altitude, latitude and longitude) and parasitoid species emerging from the beans (B = *Horismenus butcheri* 1, 2 or 3; D = *H*. *depressus*2 and M = *H*. *missourensis* 2, or 3.

Site code	Location	State	bean species of emergence	Bean species present	altitude (m)	Latitude	Longitude	HB1	HB2	HB3	HD2	HM2	HM3
CMA	Colonia Mancera	Morelos	coccineus	symp[v,c]	1730	N18 57 23.9	W98 51 47.0						
PES	Pinches Espinas	Mexico	coccineus	symp[v,c]	1737	N18 57 07.5	W99 25 17.8						
TEMS	Temescaltepec	Mexico	coccineus	symp[v,c]	1755	N19 02 26.7	W100 02 40.8	x	x				
TYN	Tlayacapan	Morelos	coccineus	symp[v,c]	1861	N18 58 28.9	W98 56 16.3						
MALS	Malinalco	Mexico	coccineus	symp[v,c]	1791–1888	N18 57 07.7	W99 30 16.5					x	x
SJS	San Jose de los Laureles	Morelos	coccineus	symp[v,c]	1830–1850	N18 58 49.6	W99 00 35.5	x	x			x	x
CVC	Cuernavaca	Morelos	coccineus	symp[v,c]	1878–1903	N18 58 41.9	W99 13 03.0	x	x			x	x
TEP	Tepoztlan	Morelos	coccineus	symp[v,c]	1885–1915	N18 59 54.6	W99 07 08.5	x	x	x	x	x	x
PB1	Pluma Blanca	Morelos	coccineus	symp[cultiv,c]	2242	N19 00 49.4	W98 56 55.2						
TLA	Tlalpan	Mexico D.F.	coccineus	allop[c]	2403	N19 17 47.9	W99 12 03.2					x	x
ATILP	Atila	Puebla	lunatus	symp[v,l]	1257	N18 36 33.9	W98 33 44.1			x	x		
YAUP	Yautepec	Morelos	vulgaris	symp[v,l]	1236	N18 55 09.0	W99 02 22.7			x	x		
TEJS	Tejupilco	Mexico	vulgaris	allop[v]	1370–1396	N18 56 04.5	W100 09 04.8	x	x				
DMSP	Dr. Miguel Silva 2	Michoacan	vulgaris	allop[v]	1478	N19 06 39.0	W101 44 21.0			x	x		
JAL	Jalmalonga	Mexico	vulgaris	symp[v,l]	1553	N18 55 01.3	W99 29 41.6	x	x				
SMG	Santa Maria Guerrero	Mexico	vulgaris	allop[cultiv,v]	1594	N18 41 36.7	W99 40 24.9						
VUL	Carretera	Morelos	vulgaris	allop[v]	1612	N18 58 12.2	W99 04 34.7			x	x		
STMS	San Martin	Mexico	vulgaris	symp[v,l]	1638	N18 55 45.3	W99 30 12.5	x	x				
CHA	Chalma	Mexico	vulgaris	symp[v,c]	1710	N18 56 56.7	W99 25 21.3						
MSILV	Dr. Miguel Silva	Michoacan	vulgaris	allop[v]	1713	N19 11 53.2	W101 44 11.7						
TEMS	Temescaltepec	Mexico	vulgaris	symp[v,c]	1755	N19 02 26.7	W100 02 40.8						
TYN	Tlayacapan	Morelos	vulgaris	symp[v,c]	1800	N18 58 02.1	W98 56 41.3						
MALS	Malinalco	Mexico	vulgaris	symp[v,c]	1791–1888	N18 57 07.7	W99 30 16.5	x	x	x	x	x	x
SJS	San Jose de los Laureles	Morelos	vulgaris	symp[v,c]	1830–1863	N18 58 49.6	W99 00 35.5			x	x	x	x
CVC	Cuernavaca	Morelos	vulgaris	symp[v,c]	1843–1903	N18 58 41.9	W99 13 03.0	x	x	x	x	x	x
TEP	Tepoztlan	Morelos	vulgaris	symp[v,c]	1885–1915	N18 59 54.6	W99 07 08.5	x	x	x	x	x	x
CAG	Cuates Aguacates	Michoacan	vulgaris	allop[v]	1910	N19 26 06.6	W101 47 50.2						
AHE	Ahuehuete	Mexico	vulgaris	symp[v,c]	2000	N18 58 21.6	W99 25 49.0						
TZINP	Tzintzintzun	Michoacan	vulgaris	allop[v]	2065	N19 38 20.9	W101 32 47.2						
JBSS	Jebús	Puebla	vulgaris	allop[v]	2164	N18 52 35.8	W98 35 59.6					x	x

To further assess the circumscription of the Mexican species of *Horismenus*, our sampling also included seven representatives of HD (collected on *Parkinsonia florida* and *Acacia gregii*) and three representatives of a yet non-described species very similar to HM (collected on *Parkinsonia microphyllum*) from Arizona (USA). In addition, *H*. *cyaeneoviridis* Girault (HC) wasps from Costa Rica were included, and molecular information from GenBank of three unidentified *Horismenus* species (*H*. sp. 1, *H*. sp. 5, *H*. sp. 6) [15] was also used. The analyses were rooted using representatives of the genus *Emersonella* (see below) using a large number of lineages.

In the full dataset, a total of 128 individuals from 14 taxa (including outgroups; see below) were selected for the analysis (Table 1). When possible, female wasps were used as they have more defined morphological features. A greater number of HB wasps were sequenced as a preliminary exploration of sequences from the first field season revealed that wasps morphologically identified as HB sorted into three separate genetic groups. As a result, additional HB wasps were added to the dataset from 2005 and 2006, and from the second field season (2008) from all available locations.

### Description of the tritropic system in Mexico

*Horismenus butcheri* is found emerging from the univoltine bruchine *Acanthoscelides obvelatus* in both *Phaseolus coccineus* and *P*. *vulgaris* beans in sympatric populations between 1843m and 1915m ([15]; Fig 1). *Horismenus depressus* is found emerging at lower altitudes (<1200m) most likely from *A*. *obtectus* and *Zabrotes subfasciatus* from both *P*. *lunatus* and *P*. *vulgaris*, as well as from *A*. *obvelatus* found in *P*. *vulgaris* populations along its entire range. In addition, where it is found to emerge from bean populations, it is almost always the most abundant wasp species. Despite emerging from populations of *P*. *vulgaris* in sympatry with *P*. *coccineus* infested by *A*. *obvelatus*, *H*. *depressus* only rarely emerges from *P*. *coccineus*. Finally, *H*. *missouriensis* attacks *A*. *obvelatus* in *Phaseolus coccineus* and *P*. *vulgaris* where they are found in sympatry as well as where they are each found alone [11, 13, 16]. This wasp species’ range extends from the highest altitudes sampled (>2400m) to more median altitudes, but does not extend into *P*. *vulgaris* populations found in sympatry with the wild lima bean, *P*. *lunatus*.

### DNA Extraction

Wasps were prepared for extraction by crushing individual wasps with a plastic pestle, with tungsten beads in a Retsch MM300 bead beater, or using non-destructive protocol by soaking overnight whole specimens in extraction buffer at 37°C (Chao-Dong Zhu, National History Museum, pers. comm.). DNA was then extracted from individuals using Qiagen’s DNeAsy Blood and Tissue extraction kit. In the non-destructive method, wasps stored in 96% EtOH were air-dried and a mix of proteinase K and Buffer ATL (Qiagen) was added. Wasps were incubated first for eight hours at 55°C, then eight hours at -20°C. This was followed by careful removal of the liquid from around the wasp and the wasps were returned to 96% EtOH. This liquid was then treated as the lysate in the remaining extraction steps of Qiagen’s DNeAsy kit.

### DNA regions

Four DNA regions were used in the analysis, two nuclear and two mitochondrial. The nuclear regions were the 28SD2 expansion region (565 bp) and ITS2 intergenic spacer (554bp) and the mitochondrial regions were 385 bp of Cytochrome B (CytB) and a 315 bp section of Cytochrome Oxidase I (COI). All *Horismenus* wasps included in the analysis were sequenced for the four gene regions. Sequences for 28SD2, ITS2 and CytB from the three unidentified *Horismenus*: *H*. sp. 1 (AY771701, AY772810 and AY820875), *H*. sp. 5 (AY771699, AY772808 and AY820872) and *H*. sp. 6 (AY771700, AY772809 and AY820873) [17] were obtained from GenBank. For the selection of outgroup taxa, the sequences of five species of *Emersonella* wasps [17] were used as they belong to the same subfamily [18]. The 28SD2, ITS2 and CytB sequences of *E*. *albicoxa* (AY771681, AY772786 and AY820845), *E*. *tanigaster* (AY771693, AY772795, and AY820869), *E*. sp. 1 (AY771690, AY772804 and AY820843), *E*. sp. 2 (AY771691, AY772805 and AY820844) and *E*. sp. 4 (AY771692, AY772801 and AY820868) [17] were also obtained from GenBank. Those *Emersonella* samples are hereafter referred to as OUT*. Representatives of the wasps for which GenBank sequences were used were provided by D. Windsor (Smithsonian Tropical Research Institute, Panama), for independent confirmation of these sequences and for COI sequencing. Due to difficulties in sequencing COI for some of these samples we used another member of the subfamily to root the tree, part of the sequence of *Entedon* (DQ149194) [19], when performing analyses of this gene alone. The 28SD2 region is known to be informative in family-level relationships in Chalcidoidea [20], and ITS2 shows a higher level of variability, which can be useful in determining relationships between genera and species. The mitochondrial genes have higher evolutionary rates and can be useful in revealing inter- and intra-species relationships.

### Amplification, DNA sequencing and alignment

To amplify the four DNA regions, the following primers were used: 28SD2 (F: 5’CGT GTT GCT TGA TAG TGC AGC3’ and R: 5’TTG GTC CGT GTT TCA AGA CGG3’) and ITS2 (F: 5’TGT GAA CTG CAG GAC ACA TG3’ and R: 5’AAT GCT TAA ATT TAG GGG GTA3’) [20, 21], CytB (F: 5’GTT CTA CTT TGA GGN CAA ATR TC3’ and R: 5’AAC TCC TAG TTT ATT NGG3’) [17] and COI (1819F: 5’GGA ACT GGA TGA ACA GTA TAT CCA CC3’ and 2191R: 5’CCA GGT AAA ATT AAA ATA TAA ACT TC3’) [22]. The PCRs were performed with final concentrations of 1X GoTaq buffer (Promega), 200uM dNTP’s, 2.0mM MgCl2, 0.5uM of both forward and reverse primers and 0.5U of GoTaq polymerase in 40ul reactions. For 28SD2 and ITS2, reactions were run with 1μl of DNA extract in a Biometra thermocycler with an initial denaturation step of 94°C for 1 minute, followed by 35 cycles of 94° for 30 seconds, 55°C for 30 seconds and 72°C for 30 seconds. A final extension of 72°C for 7 minutes was followed by a 15°C hold. For CytB, the annealing temperature was 50°C and the extension time was 60s. For COI the annealing temperature was 52°C and all steps were run for 60s. In addition, both the initial denature and final elongation steps for COI were run for 4 minutes. Five μl of each reaction were run on a 1% agarose gel to check for the presence of a product. Each successful sample was then sent to Macrogen in Seoul, South Korea where samples were purified and sequenced using either the forward or reverse primer. Sequencing was conducted under Big Dye terminator cycling conditions. The reacted products were purified using an ethanol precipitation and run using Automatic Sequencer 3730XL (Macrogen, Seoul, South Korea). Abi files created during the runs were visually scored using ChromasPro v1.5 (Technelysium Pty Ltd) and converted into a fasta format for alignment in Bioedit 7.0.9.0 [23]. Nucleotide sequences for coding genes were translated to check for pseudogenation in MEGA [24]. All sequences were aligned in Bioedit using ClustalW [25] for the initial alignment and then sequences were verified by sight. For 28SD2 and ITS2 this included assessment of gaps.

### Gap coding

The nuclear 28SD2 and ITS2 gene regions both contain indels with important information for dividing *Horismenus* into genetic groups. These indels were coded in FastGap [26] and the binary matrix was added (3 characters for 28SD2 and 111 for ITS2) to the matrix. The two coding mitochondrial DNA regions contained few indels. Included in the indel information is one long indel found in the CytB region of one of the two *H*. *cyaeneoviridis* (OB). The gap matrix was analyzed as a separate partition in the Bayesian inference (see below).

### Phylogenetic analyses

Single-gene and partitioned phylogenetic inferences were carried out employing both maximum likelihood (ML) and Bayesian Markov chain Monte Carlo (MCMC) analyses. In the case of the partitioned analyses, the dataset was divided into five partitions (each DNA region and the scored gaps) and each locus was allowed to have partition-specific model parameters. In the partitioned ML analysis the gap partition was not included since this method does not provide an evolutionary model for this type of data (see below). Computations for phylogenetic analyses were performed at the Vital-IT (http://www.vital-it.ch) Center for high-performance computing of the SIB Swiss Institute of Bioinformatics.

The ML analyses were performed using RAxML v. 8.1.11 [27, 28] with a 1000 rapid bootstrap analysis followed by the search of the best-scoring ML tree in one single run. The default model, GTRCAT, was used to perform the ML analyses. The Bayesian MCMC analyses were performed in MrBayes v.3.2 [29] and the best-fit model for each DNA region was estimated using jModeltest 0.1.1 [30] and the Akaike Information criterion (see Table 2 for best-fit models). For the protein coding genes, CytB and COI, the first two-codon positions were separated from the 3^rd^ codon position due to the vast difference in evolutionary rates.

**Table 2:** Phylogenetic markers and models of evolution. Phylogenetic markers used in the current study and models of evolution applied in Bayesian analyses of the full (128 specimens) and reduced dataset (38 specimens).

Markers	Sequence length (bp)	Gap characters	Full (128) dataset model	Reduced (38) dataset model
28SD2	565	3	K80+G	HKY+G
ITS2	554	111	HKY+G	HKY+G
CytB (1^st^ and 2^nd^ positions)	256	*	HKY+G	MT (1&2 positions)
COI (1^st^ and 2^nd^ positions)	210		K80+G	HKY+ G
CytB (3^rd^ position)	129	*	GTR+I+G	MT (3^rd^ position)
COI (3^rd^ position)	105		GTR	HKY+G

* one individual of *H*. *cyaenoviridis* has one long indel

Three Metropolis-coupled Markov chains with an incremental heating temperature of 0.2 were run for 5 million generations and sampled every 1000^th^ generation. Each analysis was repeated twice starting with random trees. The MCMC sampling was considered sufficient when the effective sampling size (ESS) was higher than 200, as verified in Tracer v1.4 [31]. After a burn-in period of 25% per run, the remaining trees were used to construct a half-compatible consensus tree (i.e., majority-rule consensus from MrBayes) and its associated Bayesian posterior probabilities (BPP).

### Species delimitation method

To support species delimitation within the Mexican *Horismenus* waps we applied the Bayesian Poisson tree process (bPTP) model developed by Zhang et al. [14] using the Bayesian half-compatible consensus tree. We followed the recommendations by Zhang et al. [14] and integrated the bPTP model with their evolutionary placement algorithm (EPA) to count the number of species on the phylogeny (bPTP-EPA). This method has been shown to outperform other methods (e.g. simple sequence similarity thresholds or OUT-picking and the General Mixed Yule coalescence model; [14]) and can be applied on non-ultrametric trees. The analysis was conducted using the portal provided by the authors (http://species.h-its.org/ptp/) based on the default parameters.

Because the bPTP-EPA method does not take phylogenetic uncertainty into account when defining the number of species, we have decided to recognize only the species that were supported by both Bayesian MCMC and ML analyses, with a Bayesian posterior probability (BPP) >0.8 and a bootstrap support >75%. Finally, we assessed the genetic identity of the species defined above by inferring their nucleotide diversities using the *nuc*.*div* function implemented in the R package *pegas* [32]. Nucleotide diversity values did not take into account of the information provided by the gaps/indels.

## Results

### Phylogenetic inference, species delimitations and nucleotide diversity

**Fig 2:**
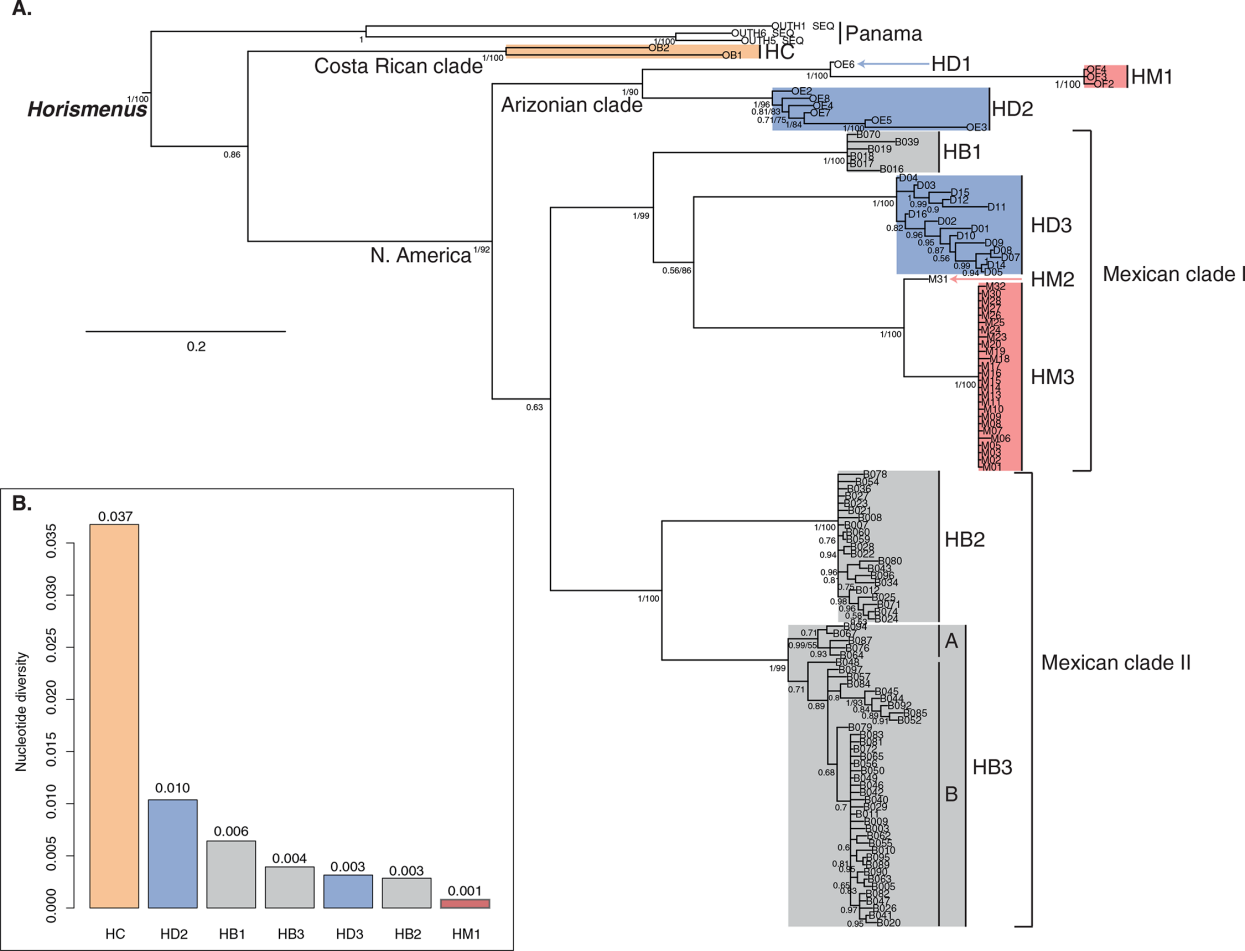
A. Combined Bayesian half-compatible consensus tree of *Horismenus* based on mitochondrial and nuclear DNA regions. The species of parasitoid wasps defined by the Bayesian Poisson tree process coupled with the evolutionary placement algorithm and node supports are represented. Bayesian posterior probabilities and bootstrap support values are displayed below branches. Please see text and Table 1 for abbreviations. **B**. Barplot of the nucleotide diversities of each species. This analysis does not include information provided by the gaps and species that are not represented have a nucleotide diversity of zero.

Although the nuclear single-gene phylogenetic trees were less resolved, no incongruences [BPP>0.8 and BS >75%] were observed between the single-gene nuclear and mitochondrial DNA phylogenetic trees allowing combining the data. The partitioned ML and Bayesian topologies were congruent in supporting the monophyly of *Horismenus* and recovering the same major clades and species delimitations within the genus (see below) and therefore only the Bayesian half-compatible consensus tree will be presented here (Fig 2A). We also favored the results of the Bayesian MCMC inference since this method allows accounting for the information provided by the scoring of the gaps/indels. The major difference between the partitioned Bayesian and ML trees laid in the resolution between clades as well as within HB3 (Mexican clade II), which supported two subclades in the former and only one in the latter (see below; Fig 2A). The nucleotide diversity analyses also allowed assessing the impact of gaps on species delimitations (see below; Fig 2B).

In this section, we will simultaneously discuss the results of the phylogenetic inference and the bPTP-EPA analysis (Fig 2A). The phylogenetic analyses supported the recognition of five clades within *Horismenus* reflecting geography: Panama clade (BPP: 1.00), Costa Rican clade (BPP: 1.00, BS: 100%), Arizonian clade (BPP: 1.00, BS: 90%), Mexican clade I (BPP: 1.00, BS: 99%) and Mexican clade II (BPP: 1.00, BS: 100%) (Fig 2A). Phylogenetic relationships between the clades are well resolved with the exception of the relationships between the two Mexican clades. With the exception of HC and the species from Panama, our phylogenetic analyses supported the polyphyly of all the species (Fig 2A). The bPTP-EPA analysis combined with node supports suggested the recognition of ten species (excluding the species from Panama) and eventually two subspecies within HB3 (Mexican clade II) (Fig 2A). As suggested by the analyses, HB might have to be split into three species (one in the Mexican clade I and two in the Mexican clade II), HD into 3 species (two in the Arizonian clade and one the Mexican clade I) and HM into 3 species (one in the Arizonian clade and two in the Mexican clade I). Finally, two species (HD1, HM2) are currently only represented by one sample and might require further investigations. With the exception of HC presenting a nucleotide diversity >0.01, all the species identified by our approach are highly homogenous (Fig 2B). The high phylogenetic clustering within HB3 is not supported by the nucleotide diversity analysis suggesting that it is provided by the gaps/indels. The same trend is observed in HM3, for which all the individuals had identical sequences.

## Discussion

The combination of these phylogenetic analyses and collection data (Fig 2) reveal that there are six species of *Horismenus* wasps that partition the local resources in Central Mexico, doubling the number of species previously known from this area. The addition of representatives of the targeted species of *Horismenus* from outside of the study area in our phylogenetic inference strongly suggests that the Mexican species of wasps are endemic to this region. Finally, we also found that the cryptic diversity in Mexican wasps has occurred, while sharing a common resource. This latter phenomenon is especially striking in the case of the *H*. *butcheri* species complex, which is shared between the two major clades (Fig 2). The species delimitations indicated by the phylogenetic analyses and biological data are strongly supported by data from the external morphology of the adult wasps. These data are from characters on the head, meso- and metasoma, and in a forthcoming paper the data will be used to define and delimit the species included here morphologically. Characters used for this purpose show little or no variation between individuals from the same clade, whereas the differences between individuals from different clades are significant. The analyses to obtain these data are in concordance with analyses of the external morphology as described by [33], the only modern and comprehensive taxonomic treatment of genus Horismenus, and the result of these analyses will be accounted for in detail in the forthcoming paper where the new species will also be formally described. Collection data shows that *H*. *butcheri* from all three genetic groups can be found emerging from the same populations, retaining their genetic identity. All the specimens assigned to the *H*. *butcheri* cryptic complex were found emerging from *A*. *obvelatus* in both *P*. *coccineus* and *P*. *vulgaris* beans in sympatric populations between 1843 and 1915m. While one of the *H*. *butcheri* clades (HB1) may be more restricted (emerging from a limited number of locations within a narrow geographical and altitudinal range, between 1843–2000m), the more closely related *H*. *butcheri* clades (HB2 and HB3) can be found together from 1370–1915 m in allopatric populations of *P*. *vulgaris* as well as sympatric populations of *P*. *vulgaris* and *P*. *coccineus*. HB3, however, is found in higher abundances and with a greater geographical range. This latter species is found across all states sampled while HB2 is concentrated more in the state of Mexico and northern and western Morelos (Table 1). While the range of wasps identified morphologically as belonging to the *H*. *butcheri* complex may cover the area sampled, abundances are quite low and wasps are mostly found at median altitudes (1500–2000m).

High geographic clustering in the phylogeny suggests a rather low dispersal rate, but it is interesting to notice that none of the species seems to be associated with a specific host plant, whereas association with a particular beetle type seems more evident (see S1 Data). How these species might have arisen remains an open question, although past geographical isolation and subsequent allopatric speciation seems a reasonable scenario, given the strong spatial structure observed here.

## Conclusions

While the phylogeny of Eulophidae [18] has been investigated for more than a decade, this is the first glimpse into the phylogenetic relationships between *Horismenus* parasitoids. The existence of several cryptic *Horismenus* entities sharing the same host on the same plant reveals high diversification rates in those parasitoid wasps associated with bruchine beetles. Given the strong fidelity of *Horismenus* wasps to specific environmental conditions [13, 15], this pattern could be associated with an ecological island syndrome [34]. The discovery of cryptic species in tropical regions has also important implications for a frequent but yet unsolved question on the prevalence of cryptic species in the tropics and the potential reasons for this pattern [35].

The information obtained in this study will not only contribute to a better insight into the diversity of tropical parasitoids, but also to their potential use as biocontrol agents. Biological control programs against crop pests are often limited by the adequate identification of the natural enemies that attack these pests. This is particularly true for tropical regions where we find higher species richness and large numbers of undescribed parasitoid species. Thus, resolving the taxonomic identity and cryptic species status of these native eulophid parasitoids, can facilitate their use as biological control agents for, bruchine beetles, pests of one of the major food staples in the world.

## Supporting Information

S1 Data Wasp Species, Host Plants and Populations sampled. The excell data includes the identity (species and sex), date of collection and origin (population and bean species) of taxa as well as number of individuals used for all analyses.
(XLSX)
